# Diagnostic accuracy of S-Detect to breast cancer on ultrasonography: A meta-analysis (PRISMA): Retraction

**DOI:** 10.1097/MD.0000000000042740

**Published:** 2025-07-18

The article, “Diagnostic accuracy of S-Detect to breast cancer on ultrasonography: A meta-analysis (PRISMA)”,^1^ which appears in Volume 101, Issue 34 of *Medicine*, is being retracted over concerns with the methodology. After publication, concerns were raised over references to the “Begger test,” which does not exist in the literature and is indicative of possible fraud. Upon further investigation, authorship irregularities were also discovered in our submission system. As a result, the Publisher no longer has confidence in the integrity of the content or the authorship.
